# Genome-wide RNAi screening identifies TMIGD3 isoform1 as a suppressor of NF-κB and osteosarcoma progression

**DOI:** 10.1038/ncomms13561

**Published:** 2016-11-25

**Authors:** Swathi V. Iyer, Atul Ranjan, Harold K. Elias, Alejandro Parrales, Hiromi Sasaki, Badal C. Roy, Shahid Umar, Ossama W. Tawfik, Tomoo Iwakuma

**Affiliations:** 1 Department of Cancer Biology, University of Kansas Medical Center, 3901 Rainbow Boulevard, Wahl East 2005, Kansas City, Kansas 66160, USA; 2 Department of Internal Medicine, Icahn School of Medicine at Mount Sinai St Luke's-Roosevelt, New York 11575, USA; 3 Department of Surgery, University of Kansas Medical Center, Kansas City, Kansas 66160, USA; 4 Department of Pathology and Laboratory Medicine, University of Kansas Medical Center, Kansas City, Kansas 66160, USA

## Abstract

The ability of cancer cells to survive and grow in anchorage- and serum-independent conditions is well correlated with their aggressiveness. Here, using a human whole-genome shRNA library, we identify TMIGD3 isoform1 (i1) as a factor that suppresses this ability in osteosarcoma (OS) cells, mainly by inhibiting NF-κB activity. Knockdown of TMIGD3 increases proliferation, tumour formation and metastasis of OS cells. Overexpression of TMIGD3 isoform1 (i1), but not isoform3 (i3) which shares a common C-terminal region, suppresses these malignant properties. Adenosine A3 receptor (A3AR) having an identical N-terminal region shows similar biological profiles to TMIGD3 i1. Protein expression of TMIGD3 and A3AR is lower in human OS tissues than normal tissues. Mechanistically, TMIGD3 i1 and A3AR commonly inhibit the PKA−Akt−NF-κB axis. However, TMIGD3 i1 only partially rescues phenotypes induced by A3AR knockdown, suggesting the presence of distinct pathways. Our findings reveal an unappreciated role for TMIGD3 i1 as a suppressor of NF-κB activity and OS progression.

## Abstract (web summary)

The ability of cancer cells to survive in anchorage-independent conditions correlates with cancer aggressiveness. Here, by screening a human whole-genome shRNA library for the ability of osteosarcoma cells to form spheres *in vitro*, the authors identify a role for TMIGD3 isoform 1 in suppressing the metastatic potential of osteosarcoma.

## Graphical abstract

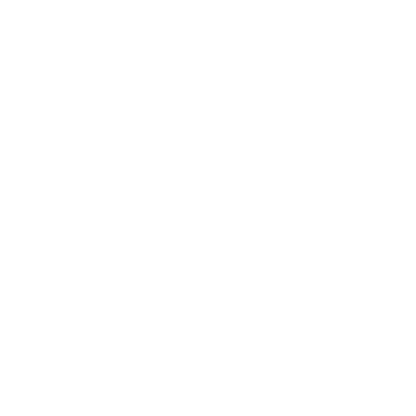

Osteosarcoma (OS) is the second leading cause of cancer-related death affecting children and adolescents^1^. Micro-metastases in OS, which eventually progress to macro-metastases, are very common at the time of diagnosis^2^. The survival rate for metastatic OS remains at 20% for the past 30 years^3^,^4^. In addition, the last two decades have seen no advances in early detection or targeted therapeutic strategies against metastatic OS^3^,^4^. This is primarily due to our limited understanding of the molecular underpinnings which drive these malignant properties in OS.

Recent advances in scientific technologies and bioinformatics have enabled unbiased genome-wide analyses to identify potential candidate genes that affect cancer-associated phenotypes. In human OS, several studies have demonstrated high occurrence of chromosome instability, the presence of susceptibility loci and altered gene expression patterns^5^,^6^,^7^,^8^. A recent whole-genome sequence analysis revealed recurrent somatic alterations in cancer genomes of paediatric OS, including translocations in the first intron of the *p53* gene^9^. In addition, a multi-stage genome-wide association study found association of a single-nucleotide polymorphism in the *Nuclear factor 1B* gene with OS metastasis^10^. Moreover, several genes and signalling pathways were identified as factors involved in OS progression via a Sleeping Beauty forward genetic screen^11^. Thus, accumulating evidence has uncovered genetic profiles and crucial factors contributing towards OS development and metastasis. Yet, the exact mechanisms underlying malignant properties of OS remain unclear.

Malignant properties of cancer cells are well correlated with their abilities to overcome cell death (anoikis: anchorage-dependent cell death) and proliferation arrest induced by loss of cell adhesion and nutritional deprivation^12^,^13^. Cancer cells that grow in these conditions can form spheres and show high tumour-forming and metastatic potential, as well as resistance to chemotherapeutic drugs^14^,^15^. However, factors that regulate sphere formation are not well understood. Identifying and characterizing these regulators would significantly advance our knowledge of molecular mechanisms behind malignant progression of cancer. We hypothesize that genes, which suppress sphere formation, would probably inhibit malignant characteristics of OS. To test this hypothesis, we have attempted to identify genes that regulate sphere-forming potential of SJSA-1 OS cells by screening a human whole-genome short hairpin RNA (shRNA) library. This screening identifies a previously uncharacterized gene, *TMIGD3* (transmembrane and immunoglobulin (Ig) domain containing 3), as a suppressor of malignant properties of OS. There are two isoforms of TMIGD3, i1 and i3, sharing all except for the first exon; only TMIGD3 i1, but not i3, plays crucial roles in suppression of malignant characteristics of OS. In addition, the first exon of *TMIGD3 i1* is shared with the first exon of *adenosine A3 receptor isoform2* (*A3AR i2*, commonly known as *A3AR*), a well-studied Gi protein-associated G-protein coupled receptor (GPCR) implicated in suppression of immunological response and tumorigenesis^16^,^17^,^18^. Protein expression of both TMIGD3 and adenosine A3 receptor (A3AR) in human OS tissues is lower than that in osteoblasts of normal bone. TMIGD3 i1 and A3AR show similar biological profiles with inhibitory effects on the PKA (protein kinase A)−Akt (or protein kinase B, PKB)−NF-κB (nuclear factor-κB) axis. However, non-overlapping functions are also present between these proteins. This is the first report demonstrating the roles of TMIGD3 i1, in the suppression of OS malignancy, as well as NF-κB activity, a commonly deregulated pathway in multiple cancers including OS.

## Results

### TMIGD3 as a factor that suppresses sphere formation

**Figure 1:**
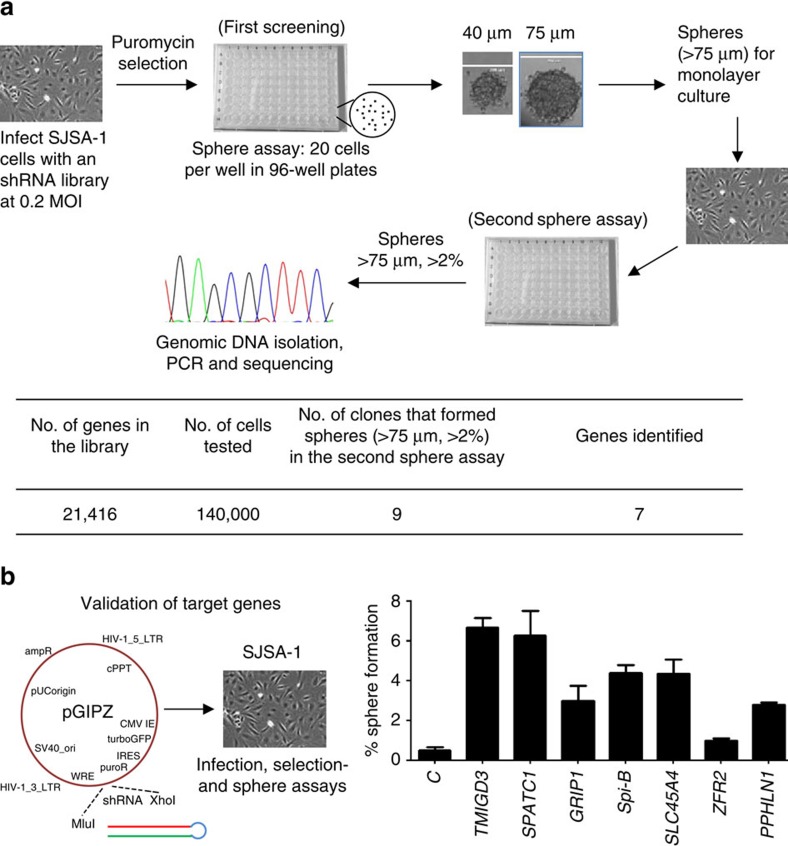
TMIGD3 as a factor that suppresses sphere formation. (**a**) Screening strategy. SJSA-1 cells infected with a human whole-genome shRNA library at 0.2 multiplicity of infection (MOI) were selected with puromycin for 48 h and subjected to sphere-formation assays (first screening) in sphere-specific conditions where 20 cells per well were plated in 96-well ultra-low attachment plates in serum-free sphere media. Sizes of spheres were determined 2 weeks later and spheres with sizes >75 μm in diameter were isolated and expanded in monolayer culture. These sphere-derived cells were further subjected to secondary sphere assays (second sphere assay), where sphere-derived clones that formed spheres >75 μm at >2% of frequency were further analysed for identification of the respective shRNA using genomic PCR and sequencing. Summary of screening results is presented below the schematic strategy. Our sequencing results of nine clones identified seven genes, as three of the nine spheres had the same shRNA against the gene *TMIGD3*. (**b**) Validation of candidate genes. The pGIPZ lentiviral vectors containing each identified shRNA for seven genes were infected into SJSA-1 cells, followed by puromycin selection and sphere-formation assays. Total number of cells examined was 2,000 per clone. Graph showing percentage of sphere formation (a percentage of number of spheres formed/number of cells seeded). Error bars: means±s.d. (*n*=3 independent experiments).

We attempted to identify factors that could regulate sphere formation of OS cells through a genome-wide screening. First, to determine an appropriate cell line to be used for screening, we tested sphere-forming potential of several OS cell lines, including U2OS, Saos2, SJSA-1, MG63 and KHOS/NP cells, in anchorage- and serum-independent sphere-specific conditions in ultra-low attachment 96-well plates. We seeded 20 cells per well so that each sphere was of a clonal origin and not derived from aggregates. To measure the sphere-forming potential, we counted spheres with sizes >30 μm in diameter and categorized the formed spheres into two groups by sizes: 30–75 μm and >75 μm. We observed no sphere formation from U2OS and Saos2 cells, low sphere-forming potential in SJSA-1 cells and high potential in MG63 and KHOS/NP cells (Supplementary Table 1). We chose SJSA-1 cells for further screening purposes, as it retained the ability to form spheres and sizes of all spheres were <75 μm, thus having a low background. SJSA-1 cells were then infected with a human whole-genome shRNA lentiviral vector library at 0.2 multiplicity of infection, so that only a single shRNA was present per cell resulting in downregulation of a single gene/factor, which would contribute towards increased sphere-forming potential. After selection with puromycin, the infected cells were subjected to sphere-formation assays (first screening, Fig. 1a). Spheres formed with sizes >75 μm in diameter were isolated, cultured for expansion and then subjected to secondary sphere-formation assays, to further confirm their increased sphere-forming potential and to rid false positives (second sphere assay, Fig. 1a). Nine clones were selected, as they formed spheres >75 μm in diameter with a frequency of >2%, which was significantly higher than that of parental SJSA-1 cells (<1%, Supplementary Table 1). These nine clones were expanded, followed by genomic DNA isolation. PCR was performed using specific PCR primers flanking the shRNA site and the PCR products were further sequenced to identify the target sequences within shRNAs (Fig. 1a). Our sequencing results of nine clones identified seven genes (Supplementary Table 2). Three of the nine spheres had the same shRNA against the gene *TMIGD3*. To further validate the effects of these seven genes/factors on sphere-forming potential of SJSA-1 cells, we infected SJSA-1 cells with lentiviral vectors encoding each shRNA identified for these seven genes (Fig. 1b). Knockdown of all the genes, except *zinc finger RNA binding protein 2 (ZFR2)*, recapitulated the sphere-forming ability and satisfied the criteria (>75 μm, >2%). Of these, SJSA-1 cells infected with lentiviral vectors encoding shRNAs for *TMIGD3* and *spermatogenesis and centriole associated 1* (*SPATC1*) showed the highest sphere-forming abilities (Fig. 1b). As three clones contained the shRNA for *TMIGD3*, we decided to further characterize *TMIGD3* for its role in OS progression.

### TMIGD3 knockdown enhances malignancy of OS cells

**Figure 2:**
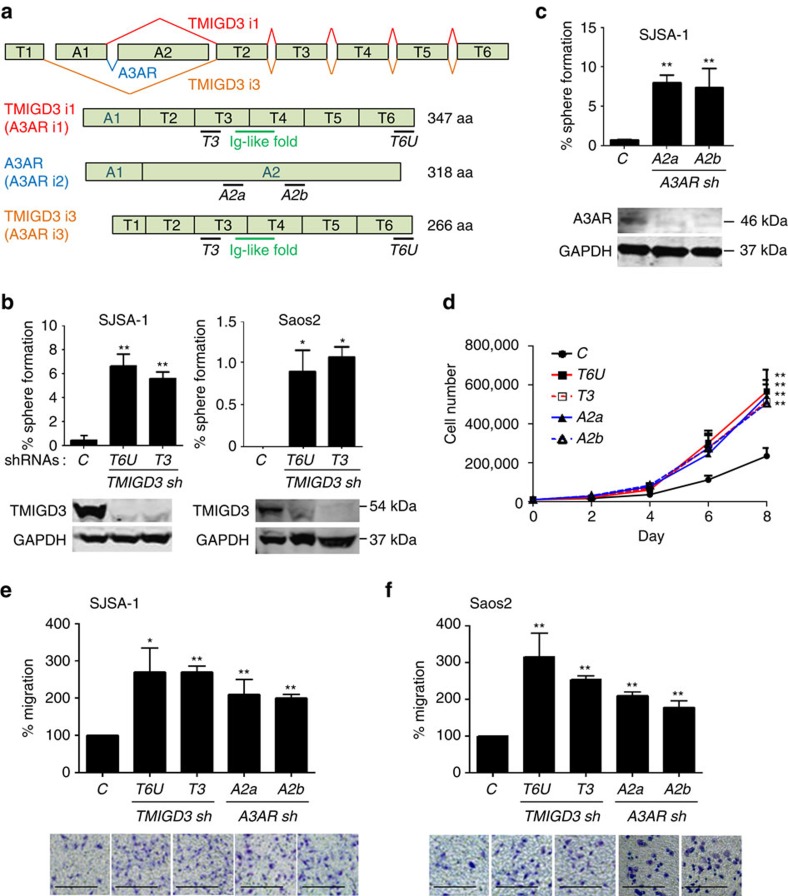
TMIGD3 knockdown increases malignant properties of OS cells. (**a**) Human *TMIGD3* gene locus on chromosome 1 and gene structure of human *TMIGD3 i1* (*A3AR i1*, red), *A3AR* (*A3AR i2*, blue) and *TMIGD3 i3* (*A3AR i3*, orange). Black bars: locations of target sequences of each shRNA: *T6U*, *T3* (targeting TMIGD3 i1 and i3); *A2a*, *A2b* (targeting A3AR). Green bar: Ig-like fold. (**b**,**c**) Sphere-formation assays using SJSA-1 and/or Saos2 cells with different shRNAs for TMIGD3 (**b**) and A3AR (**c**). Graphs showing percentage of sphere formation. Error bars: means±s.d. (*n*=1,440 from 3 independent experiments). *P<0.05 and **P<0.01; Student's *t*-test (two-tailed). (**d**) Proliferation assays following knockdown of TMIGD3 (*T6U*, *T3*) and A3AR (*A2a*, *A2b*) in SJSA-1 cells. Cells (1 × 10^4^) were seeded on six-well plates and numbers of cells were counted every 2 days following trypan blue staining. Error bars: means±s.d. (*n*=3 independent experiments). **P<0.01, two-way analysis of variance. (**e**,**f**) Transwell migration assays for 10 h using SJSA-1 (**e**) and Saos2 (**f**) cells downregulated for TMIGD3 or A3AR. Graphs showing relative migration to the control (top) and representative images (below). Error bars: means±s.d. (*n*=3 independent experiments). *P<0.05 and **P<0.01; Student's *t*-test (two-tailed). Scale bar, 25 μm. Unprocessed original scans of blots for **b**,**c** are shown in Supplementary Fig. 7.

TMIGD proteins are a group of proteins that contain an ‘Ig-like fold'. TMIGD1 is implicated in cell differentiation and adhesion^19^,^20^, whereas TMIGD2 is implicated in cancer immunosuppression as a receptor of HHLA2, a B7 family member^21^. However, there is no previous report about TMIGD3. TMIGD3 comprises of two identified isoforms: i1 and i3, with exclusive first exons (A1/T1), whereas their carboxy-terminal region (exons T2–T6) is overlapped (Fig. 2a). Intriguingly, there is a splicing variant for TMIGD3 i1 that shares the first exon (A1), which is well characterized as A3AR, one of the four adenosine receptors with a typical seven-transmembrane helical structure of a GPCR and is implicated in the suppression of several cancers and autoinflammatory diseases^16^. Thus, TMIGD3 i1 is sometimes referred to as A3AR i1, whereas A3AR is precisely A3AR isoform2 (i2). Although TMIGD3 i3 does not have any overlapping region with A3AR, it is still occasionally called as A3AR i3, probably because these genes are present in the same chromosomal locus (Fig. 2a). To avoid confusion, we followed TMIGD3 i1 and i3 nomenclature, instead of using A3AR i1 and i3, as these proteins share an Ig-like domain in the common C-terminal region and their functions as adenosine receptors are unknown.

Our identified shRNA for *TMIGD3* (*T6U*) was located in the 3′-untranslated region (UTR), which targets both TMIGD3 i1 and i3, but not A3AR. To further confirm the significance of TMIGD3 in the malignant properties of OS, we used another shRNA (*T3*) to downregulate the expression of TMIGD3 (Fig. 2a). Successful knockdown of TMIGD3 by *T6U* and *T3* shRNAs was confirmed by western blotting using our generated PAb128 antibody, a peptide antibody against amino acid (aa) sequences in the exon T3 (Fig. 2b and Supplementary Fig. 1a). Following TMIGD3 knockdown, we observed increase in sphere formation of SJSA-1, Saos2 and MG63 OS cells (Fig. 2b and Supplementary Fig. 1b). As TMIGD3 i1 shares its amino-terminal 117aa with A3AR and the role of A3AR in the suppression of other cancers is established, we wanted to query the importance of A3AR in the sphere-forming ability of OS cells. Knockdown of A3AR with two different shRNAs (*A2a* and *A2b*) increased the sphere-forming ability of SJSA-1 (Fig. 2c), as well as Saos2 and MG63 cells (Supplementary Fig. 1c,d).

Knockdown of TMIGD3 or A3AR also increased cell proliferation of SJSA-1 cells (Fig. 2d). In addition, knockdown of TMIGD3 or A3AR enhanced migration of SJSA-1 and Saos2 cells (Fig. 2e,f). These results suggest that both TMIGD3 and A3AR could play roles in suppressing malignant progression of OS cells.

### Association of reduced TMIGD3 or A3AR with OS malignancy

**Figure 3:**
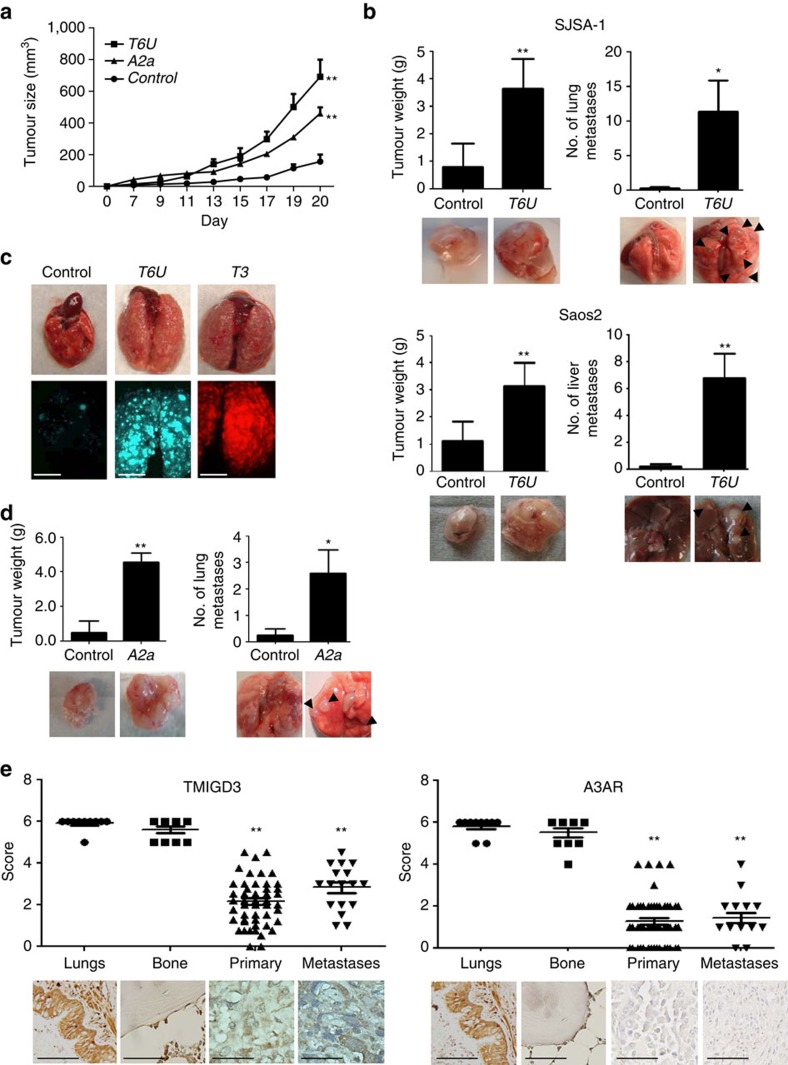
Association of reduced TMIGD3 or A3AR with OS malignancy. (**a**) Subcutaneous tumour formation assays using SJSA-1 cells (5 × 10^5^) downregulated for non-silencing shRNA (Control), A3AR (*A2a*) or TMIGD3 (*T6U*). Tumours were measured twice a week until day 20 after injections. Error bars: means±s.d. (*n*=5 mice per group). **P<0.01; two-way analysis of variance (ANOVA). (**b**) Primary tumours and metastases following orthotopic injections of SJSA-1 (top, *n*=5 mice per group) and Saos2 (bottom, *n*=5 mice per group) cells with or without TMIGD3 knockdown (*T6U*). Cells (1 × 10^5^) were injected into femurs of NOD-scid IL2Rγ^null^ (NSG) mice. Mice were killed ∼2 months (SJSA-1) or 5 months (Saos2) later, when thigh diameter became ∼2 cm in mice injected with TMIGD3 knockdown cells. Graphs showing primary tumour weight and number of metastatic nodules in the lungs (SJSA-1) or livers (Saos2). Representative images of primary tumours and metastases (arrows) below the graphs. Error bars: means±s.d. *P<0.05 and **P<0.01; Student's *t*-test (two-tailed). (**c**) Tail vein injection assays using SJSA-1 cells (5 × 10^4^) infected with non-silencing (control: GFP+, green), *T6U* (GFP+, green) or *T3* (DsRed+, red) shRNAs. Mice (Control*: n*=7, *T6U*: *n*=7, *T3: n*=4) were killed 6 weeks later. Representative pictures of lungs (top) and images from a fluorescence dissecting stereo-microscope (bottom). Scale bar, 5 mm. (**d**) Primary tumours and metastases (arrows) following orthotopic injections of SJSA-1 cells (1 × 10^5^) with or without A3AR knockdown. Mice were monitored for ∼2 months. Graphs showing primary tumour weight and number of metastatic nodules in the lungs. Representative images of the primary tumours and metastatic nodules below the graphs. Error bars: means±s.d. (*n*=5 mice per group). *P<0.05 and **P<0.01; Student's *t*-test (two-tailed). (**e**) Immunohistochemistry (IHC) for TMIGD3 (left) and A3AR (right) in human OS and normal tissues. IHC analyses were performed independently in OS tissues (*n*=54 primary tissues and 17 metastases), as well as normal lung (*n*=10) and bone (*n*=10) tissues. Scoring was done on a scale of 0–6. Representative images of IHC are below the graphs. The horizontal lines in the plots represent the median. **P<0.01; one-way ANOVA. Scale bar, 50 μm.

To further address the effects of TMIGD3 and A3AR on OS malignancy *in vivo*, we performed subcutaneous tumour growth assays following knockdown of TMIGD3 or A3AR. We observed that downregulation of TMIGD3 or A3AR led to increase in tumour growth in SJSA-1 cells (Fig. 3a). Interestingly, concomitant knockdown of TMIGD3 and A3AR showed a cooperative effect on tumour growth (Supplementary Fig. 2a). We further performed orthotopic tumour transplantation assays by injecting SJSA-1 and Saos2 cells with or without knockdown of TMIGD3 into the femurs of immunocompromised NOD-scid IL2Rγ^null^ mice^22^. Knockdown of TMIGD3 significantly enhanced primary tumour growth in femurs and metastasis formation in the lungs (SJSA-1) or the livers (Saos2, Fig. 3b). As TMIGD3 knockdown increased metastasis, we also performed tail vein injection assays to test the ability of tumour cell colonization in the lungs. Consistently, SJSA-1 cells with knockdown of TMIGD3 by *T6U* or *T3* shRNAs dramatically increased lung metastases (Fig. 3c). Similarly, knockdown of A3AR (*A2a*) in SJSA-1 cells also enhanced tumour growth and lung metastasis in orthotopic injection assays, although metastases were not as robust as TMIGD3 knockdown (Fig. 3d).

To further address the clinical significance of TMIGD3 and A3AR in OS, we examined the expression levels of these proteins in human tissues. We first validated our generated TMIGD3 antibody (PAb128) for the use of immunohistochemistry (IHC) using SJSA-1-derived tumour tissues with or without TMIGD3 knockdown. Membrane distribution of TMIGD3 protein was observed in the control tumour, whereas in the TMIGD3-knockdown tumour (*T6U*) the staining intensity was low, thus confirming the specificity of the PAb128 antibody (Supplementary Fig. 2b). Using this antibody and a commercially available A3AR antibody, we performed IHC for these proteins in primary and metastatic human OS tissues, as well as normal lung and bone tissues as controls. Strong cytoplasmic staining of both TMIGD3 and A3AR was observed in the bronchial cells in normal lungs and osteoblasts in normal bone tissues (Fig. 3e). However, levels of both TMIGD3 and A3AR were significantly reduced in both primary and metastatic human OS tissues. We did not find any significant difference in the expression of both TMIGD3 and A3AR between primary and metastatic OS. These data suggest that reduced expression of TMIGD3 or A3AR is associated with malignant properties of OS *in vivo* and clinically.

### Inhibition of OS malignancy by TMIGD3 i1 and A3AR but not i3

**Figure 4:**
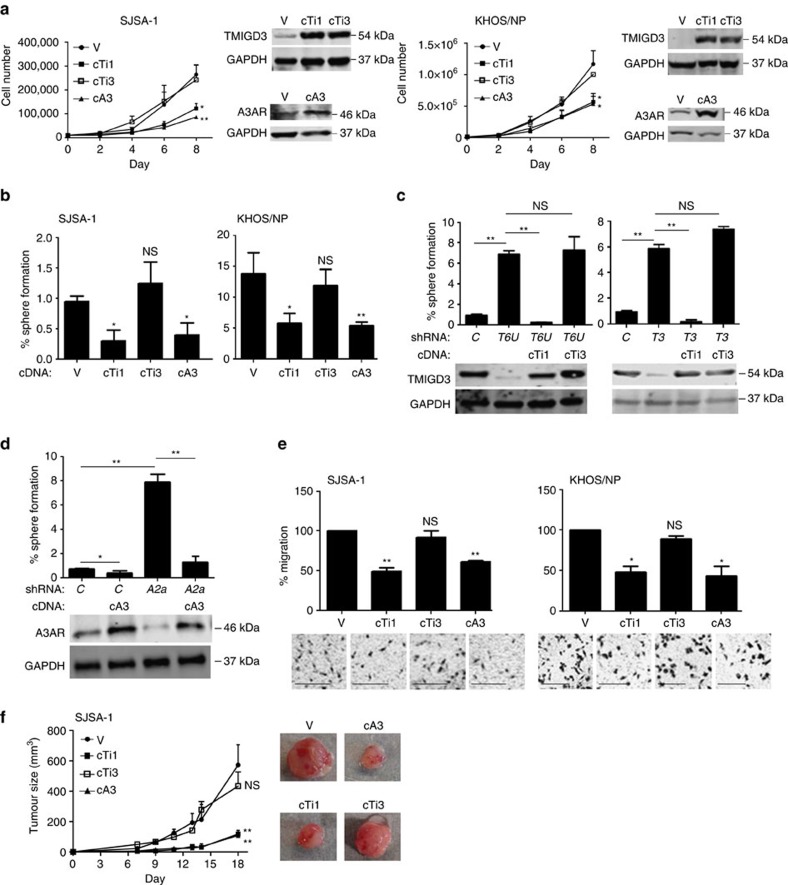
Inhibition of OS malignancy by TMIGD3 i1 and A3AR but not i3. (**a**) Proliferation assays using SJSA-1 (left) and KHOS/NP (right) cells overexpressing vector control (V), TMIGD3 i1 cDNA (cTi1), TMIGD3 i3 cDNA (cTi3) or A3AR cDNA (cA3). Representative immunoblots showing overexpression of each protein next to the graphs. Error bars: means±s.d. (*n*=3 independent experiments).*P<0.05 and **P<0.01; two-way analysis of variance (ANOVA). (**b**) Sphere-formation assays using SJSA-1 and KHOS/NP cells overexpressing V, cTi1, cTi3 or cA3. Graph showing % of sphere formation. Error bars: means±s.d. (*n*=3 independent experiments). *P<0.05 and **P<0.01, NS, not significant; Student's *t*-test (two-tailed). (**c**) Sphere-formation assays using SJSA-1 cells with or without knockdown of TMIGD3 (*T6U*, *T3*) along with overexpression of TMIGD3 i1 (cTi1) or i3 (cTi3). Representative immunoblots below the graphs. Error bars: means±s.d. (*n*=3 independent experiments). **P<0.01; NS, not significant; Student's *t*-test (two-tailed). (**d**) Sphere-formation assays using SJSA-1 cells with overexpression of A3AR (cA3), knockdown of A3AR (*A2a*) or knockdown of A3AR (*A2a*) along with overexpression of A3AR (cA3). Representative immunoblots are below the graphs. Error bars: means±s.d. (*n*=3 independent experiments). *P<0.05 and **P<0.01; Student's *t*-test (two-tailed). (**e**) Transwell migration assays for 10 h using SJSA-1 (left) and KHOS/NP (right) cells overexpressing V, cTi1, cTi3 or cA3. Graphs showing relative migration to the control (top) and representative images (below). Error bars: means±s.d. (*n*=3 independent experiments). *P<0.05 and **P<0.01; NS, not significant; Student's *t*-test (two-tailed). Scale bar, 25 μm. (**f**) Tumour growth assays in mice injected with SJSA-1 cells overexpressing V, cTi1, cTi3 or cA3. Cells (1 × 10^6^) were subcutaneously injected into nude mice, and tumour sizes were monitored twice a week for 18 days. Error bars: means±s.d. (*n*=5 mice per group). Representative images of tumours are next to the graph. **P<0.01; NS, not significant; two-way ANOVA.

*TMIGD3* has two isoforms (i1 and i3) that share the C-terminal region containing the Ig-like fold (Fig. 2a). As the shRNAs used in our study (*T6U* and *T3*) target both TMIGD3 i1 and i3, it remains unclear which isoform(s) are responsible for phenotypes associated with OS malignancy following TMIGD3 knockdown. To address this concern and further compare their biological phenotypes with that of A3AR, we stably infected lentiviral vectors encoding complementary DNAs (cDNA, coding region) for TMIGD3 i1 (cTi1), TMIGD3 i3 (cTi3) and A3AR (cA3), as well as empty vector (V), in SJSA-1 and KHOS/NP cell lines. Cell proliferation assays showed that overexpression of TMIGD3 i1 and A3AR, but not TMIGD3 i3, significantly inhibited proliferation of both the cell lines (Fig. 4a). Intriguingly, western blotting for these proteins revealed that both TMIGD3 i1 and TMIGD3 i3 ran at almost the same size at ∼54 kDa, higher than their predicted sizes of ∼40 kDa and ∼30 kDa, respectively (Fig. 4a). To validate this, we performed western blotting using cells transfected with Flag-tagged TMIGD3 i3 (Fl-cTi3), together with non-tagged TMIGD3 i1 and i3, and found that even Flag-tagged TMIGD3 i3 ran at a similar size to TMIGD3 i1 and i3 (Supplementary Fig. 3a). It should be noted that similar differences in predicted size versus actual size have been observed previously for both TMIGD1 and TMIGD2, due to posttranslational modifications such as *N*-glycosylation^19^,^23^. We then performed sphere-formation assays using SJSA-1, KHOS/NP and MG63 cells following overexpression of TMIGD3 i1, TMIGD3 i3 or A3AR. KHOS/NP and MG63 cells are highly aggressive OS cell lines with high sphere-forming potential (Supplementary Table 1). In accordance with the proliferation assay results, TMIGD3 i1 and A3AR, but not TMIGD3 i3, significantly suppressed sphere formation of SJSA-1 and KHOS/NP cell lines (Fig. 4b). Similarly, TMIGD3 i1 and A3AR both suppressed sphere formation of MG63 cells (Supplementary Fig. 3b). These results suggest that TMIGD3 i1, but not i3, suppresses malignant properties of OS, similar to A3AR.

We next wanted to address possible off-target effects of used shRNAs and examine whether overexpression of TMIGD3 i1 or TMIGD3 i3 could rescue increased sphere formation of SJSA-1 cells by TMIGD3 knockdown. Overexpression of TMIGD3 i1 successfully nullified the increased sphere formation by *T6U* and *T3* shRNAs, whereas TMIGD3 i3 overexpression failed to do so (Fig. 4c). Similarly, overexpression of A3AR cDNA (coding region) cancelled the increased sphere formation by the *A2a* shRNA (Fig. 4d). Of note, although A3AR overexpression sufficiently rescued protein levels of A3AR, the *A2a* shRNA could still target exogenously expressed A3AR (Fig. 4d). Hence, we set up another shRNA targeting the 3′-UTR of *A3AR* (*A2U*). Increased sphere formation of SJSA-1 cells by the *A2U* shRNA was successfully rescued by overexpression of A3AR coding region (Supplementary Fig. 3c). In addition, we also set up a small interfering RNA (siRNA) that spans the splicing junction of exons A1 and T2 (*ATa*), and found that the *ATa* siRNA successfully reduced TMIGD3 i1 alone with minimal effects on TMIGD3 i3 and A3AR in KHOS/NP cells that were overexpressed for TMIGD3 i1, TMIGD3 i3 or A3AR, as KHOS/NP cells endogenously expressed low levels of these proteins (Supplementary Fig. 3d). Knockdown of TMIGD3 i1 by the *ATa* siRNA consistently increased sphere formation of SJSA-1 cells (Supplementary Fig. 3e). These results strongly corroborate the observed increase in sphere formation by shRNAs targeting TMIGD3 and A3AR, and eliminate possible off-target effects.

In addition, overexpression of TMIGD3 i1 and A3AR, but not TMIGD3 i3, inhibited migration of SJSA-1 and KHOS/NP cells (Fig. 4e), as well as tumour growth of SJSA-1 cells (Fig. 4f), thereby suggesting that TMIGD3 i1 and A3AR suppress malignant properties of OS cells *in vitro* and *in vivo*.

### Regulation of the NF-κB pathway by TMIGD3 i1 and A3AR

**Figure 5:**
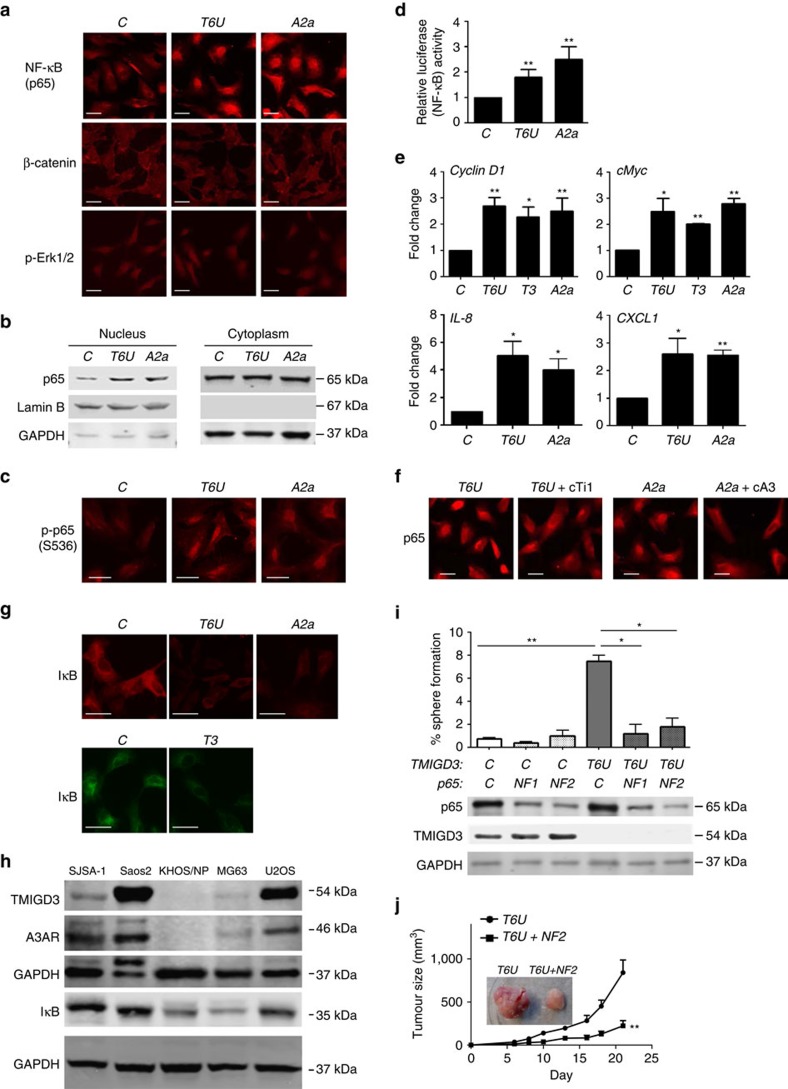
Regulation of the NF-κB pathway by TMIGD3 i1 and A3AR. (**a**) Immunofluorescence for NF-κB (p65), β-catenin and p-Erk1/2 using SJSA-1 cells infected with lentiviral vectors encoding non-silencing control (*C*), *T6U* (TMIGD3) or *A2a* (A3AR) shRNAs. Scale bar, 50 μm. (**b**) Immunoblots for p65, Lamin B and glyceraldehyde 3-phosphate dehydrogenase (GAPDH), using nuclear and cytoplasmic extracts of SJSA-1 cells with or without downregulation of TMIGD3 (*T6U*) or A3AR (*A2a*). (**c**) Immunofluorescence for phosphorylated p65 (p-p65) at serine 536 (S536) using SJSA-1 cells with or without downregulation of TMIGD3 or A3AR. Scale bar, 50 μm. (**d**) Luciferase assays for measuring the NF-κB activity in SJSA-1 cells downregulated for TMIGD3 or A3AR. Graph showing relative luciferase activity normalized to that of SJSA-1 cells infected with non-silencing control lentiviral vector. Error bars: means±s.d. (*n*=3 independent experiments). **P<0.01; Student's *t*-test (two-tailed). (**e**) Quantitative reverse transcription–PCR for *cyclin D1*, *cMyc*, *IL-8* and *CXCL1* using SJSA-1 cells downregulated for TMIGD3 (*T6U*, *T3*) or A3AR (*A2a*). Relative mRNA expression was standardized to that of *GAPDH* and normalized by values in non-silencing control vector-infected cells. Error bars: means±s.d. (*n*=3 independent experiments). *P<0.05 and **P<0.01; Student's *t*-test (two-tailed). (**f**) Immunofluorescence for p65 using SJSA-1 cells downregulated for TMIGD3 (*T6U*) or A3AR (*A2a*) without or with overexpression of TMIGD3 i1 (cTi1) or A3AR (cA3), respectively. Scale bar, 50 μm. (**g**) Immunofluorescence for IκB using SJSA-1 cells downregulated for TMIGD3 (*T6U*, *T3*) or A3AR (*A2a*). Scale bar, 50 μm. (**h**) Immunoblots for indicated proteins using different OS cell lines. (**i**) Sphere-formation assays using SJSA-1 cells with or without knockdown of TMIGD3 and/or NF-κB/p65 (*NF1*, *NF2*). Representative immunoblots for indicated proteins below the graph. Error bars: means±s.d. (*n*=3 independent experiments). *P<0.05 and **P<0.01; Student's *t*-test (two-tailed). (**j**) Subcutaneous tumour formation assays using SJSA-1 cells with knockdown of TMIGD3 (*T6U*) and/or p65 (*NF2*). Graph and inset showing tumor size (mm^3^) and representative images of tumours at day 21, respectively. Error bars: means±s.d. (*n*=5 mice per group). **P<0.01; two-way ANOVA. Unprocessed original scans of blots for **b**,**h** are shown in Supplementary Fig. 7.

Our results demonstrated similar biological profiles between TMIGD3 i1 and A3AR. To mechanistically understand functional similarity of TMIGD3 i1 with A3AR, we first performed web-based domain analyses using protein structure prediction sites, including PredictProtein server (https://www.predictprotein.org/) and SOSUI (http://harrier.nagahama-i-bio.ac.jp/sosui/sosui_submit.html), which identified two transmembrane helices at the C-terminal region of TMIGD3 i1 (aa129–151 and aa291–313), in addition to 3 transmembrane helices within the first 117 amino acids shared with A3AR, as well as a known Ig-like fold at the C-terminal region (aa167–256, Supplementary Fig. 4). Given that A3AR is a GPCR with seven-transmembrane helices^24^ and TMIGD3 i1 preserves a part of the A3AR structure, we questioned whether TMIGD3 i1 could regulate signalling similar to A3AR, which is known to inhibit three major cancer-associated signalling including the NF-κB, β-catenin and Erk pathways (Supplementary Fig. 5a)^25^,^26^. To address this, we examined cellular localization of NF-κB (p65), β-catenin and p-Erk1/2, as nuclear localization of these proteins is well correlated with their activities. We observed that knockdown of TMIGD3 (*T6U*) significantly increased nuclear localization of p65 with minimal effects on β-catenin and p-Erk1/2 localization in SJSA-1 cells, similar to A3AR knockdown by the *A2a* shRNA (Fig. 5a). We also confirmed minimal effects of TMIGD3 and A3AR knockdown on the β-catenin activities by TOPFlash T-cell factor/lymphoid enhancer factor (TCF/LEF)-responsive firefly luciferase reporter assays (Supplementary Fig. 5b). We further confirmed increased NF-κB activity by p65 accumulation in the nuclear fraction (Fig. 5b), increase in p65 phosphorylation at serine 536 (Fig. 5c), increase in the NF-κB transcriptional activity through luciferase reporter assays (Fig. 5d) and increased messenger RNA transcript levels of NF-κB downstream targets *cyclin D1*, *cMyc*, *IL-8* and *CXCL1* (Fig. 5e), following knockdown of TMIGD3 or A3AR in SJSA-1 cells. In addition, increased nuclear localization of p65 in SJSA-1 cells by knockdown of TMIGD3 (*T6U*) or A3AR (*A2*a) was abrogated by overexpression of TMIGD3 i1 (cTi1) or A3AR (cA3), respectively (Fig. 5f), suggesting that increase in the NF-κB activities by the *T6U* and *A2a* shRNAs were not due to their off-target effects. In addition, immunofluorescence studies revealed reduced levels of IκB, an inhibitor of NF-κB, following knockdown of TMIGD3 or A3AR (Fig. 5g). These results suggest that both TMIGD3 i1 and A3AR may suppress malignant properties of OS by inhibiting NF-κB activity.

To examine a possible correlation of expression levels of TMIGD3 or A3AR with NF-κB activity in different OS cell lines, we performed western blotting for TMIGD3, A3AR and IκB. We observed that U2OS, Saos2 and SJSA-1 cells with low sphere-forming potential had high levels of TMIGD3 and A3AR, as well as IκB, whereas MG63 and KHOS/NP cells with high sphere-forming potential had low levels of these proteins (Fig. 5h and Supplementary Table 1).

We next examined whether enhanced malignancy associated with TMIGD3 knockdown could be rescued by simultaneous knockdown of NF-κB/p65. Indeed, concomitant knockdown of p65 attenuated sphere formation and subcutaneous tumour growth of SJSA-1 cells enhanced by TMIGD3 knockdown (Fig. 5i,j). It should be noted that the observed effects of p65 knockdown were partial and not complete. These data suggest that suppressive effects of TMIGD3 i1 and A3AR on the malignant properties of OS cells are regulated mainly, but not solely, through inhibition of NF-κB activity.

### Distinct pathways regulated by TMIGD3 i1 and A3AR

**Figure 6:**
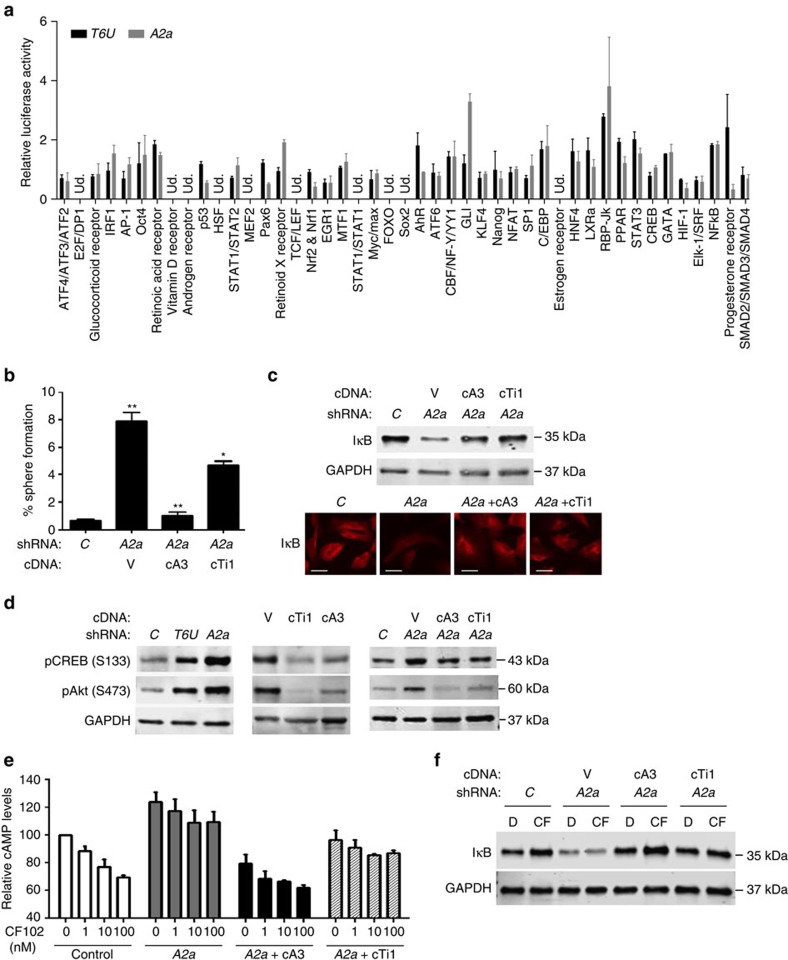
Non-overlapping pathways regulated by TMIGD3 i1 and A3AR. (**a**) Luciferase-based signal array experiments using SJSA-1 cells downregulated for TMIGD3 (*T6U*, black) or A3AR (*A2a*, grey). Graph showing relative luciferase activity normalized to that of SJSA-1 cells infected with non-silencing control lentiviral vector. Ud., undetectable. Error bars: mean±s.d. (*n*=3 independent experiments). (**b**) Sphere-formation assays using SJSA-1 cells with or without knockdown of A3AR (*A2a*), with concomitant overexpression of A3AR (cA3) or TMIGD3 i1 (cTi1). Graph represents sphere-forming potential. Error bars: means±s.d. (*n*=3 independent experiments). *P<0.05 and **P<0.01; Student's *t*-test (two-tailed). (**c**) Immunoblots (top) and immunofluorescence (bottom) for IκB and glyceraldehyde 3-phosphate dehydrogenase (GAPDH) using SJSA-1 cells downregulated for A3AR with or without overexpression of A3AR (cA3) or TMIGD3 i1 (cTi1). Scale bar, 50 μm. (**d**) Immunoblots for phosphorylated CREB (pCREB) at S133, phosphorylated Akt (pAkt) at S473 and GAPDH, using SJSA-1 cells with knockdown for non-silencing control (*C*), TMIGD3 (*T6U*) or A3AR (*A2a*, left), overexpression of vector control (V), TMIGD3 i1 (cTi1) or A3AR (cA3, middle) and knockdown for A3AR plus overexpression of A3AR (cA3) or TMIGD3 i1 (cTi1) (right). (**e**) Relative cAMP levels in SJSA-1 cells with or without knockdown of A3AR (*A2a*) plus overexpression of A3AR (cA3) or TMIGD3 i1 (cTi1) treated with different concentrations of an A3AR-specific agonist, CF102. The graph represents relative cAMP levels compared with control SJSA-1 cells treated with dimethyl sulfoxide (DMSO). Error bars: means±s.e.m. (*n*=3 independent experiments). (**f**) Immunoblots for IκB and GAPDH using SJSA-1 cells downregulated for A3AR with or without overexpression of A3AR (cA3) or TMIGD3 i1 (cTi1), following treatment with DMSO (D) or CF102 (CF, 100 nM) for 16 h. Unprocessed original scans of blots for **d** (middle panel) are shown in Supplementary Fig. 7.

Our data suggest that suppressive effects of TMIGD3 and A3AR on the malignant properties of OS cells could be regulated by differential pathways other than the NF-κB pathway. We hence compared signalling pathways altered by knockdown of either TMIGD3 or A3AR through an unbiased luciferase-based signal array experiment in SJSA-1 cells (Fig. 6a). First, we noted that knockdown of TMIGD3 or A3AR consistently showed increase in the NF-κB activity. Second, the activities of TCF/LEF (transcription factors for β-catenin signalling) and Elk-1 (downstream of Erk signalling) were either undetectable or unaffected by knockdown of TMIGD3 or A3AR, supporting our results in Fig. 5a. Finally, there were distinct pathways altered by knockdown of these proteins (Fig. 6a).

We next inquired whether TMIGD3 i1 compensated for increase in sphere formation by A3AR knockdown. Overexpression of TMIGD3 i1 (cTi1) only partially suppressed the increased sphere formation of SJSA-1 cells by A3AR knockdown, whereas A3AR overexpression (cA3) nullified it (Fig. 6b). These data further suggest that A3AR could regulate sphere-forming potential via both overlapping and non-overlapping pathways with TMIGD3 i1.

We then attempted to investigate whether or not TMIGD3 i1 could inhibit upstream signalling of NF-κB similar to A3AR. We first examined the effect of TMIGD3 i1 overexpression on IκB levels in SJSA-1 cells downregulated for A3AR. Western blotting and immunofluorescence studies revealed that reduced IκB levels following A3AR knockdown were significantly restored on TMIGD3 i1 overexpression, similar to A3AR overexpression (Fig. 6c). We next examined the effects of TMIGD3 i1 on activities of PKA and Akt, known upstream regulators of IκB and downstream effectors of A3AR. The activities of PKA and Akt were assessed by measuring phosphorylation status of CREB at serine 133 (S133) and Akt at serine 473 (S473), respectively. Knockdown of TMIGD3 or A3AR resulted in increased phosphorylation of CREB and Akt (Fig. 6d, left), whereas overexpression of TMIGD3 i1 or A3AR showed reduction in the phosphorylation of these two proteins (Fig. 6d, middle). Moreover, increased phosphorylation of CREB and Akt in A3AR knockdown cells was significantly attenuated when either A3AR or TMIGD3 i1 was overexpressed (Fig. 6d, right). Thus, TMIGD3 i1 regulates the PKA−Akt−IκB−NF-κB axis, similar to A3AR, thus reiterating the significance of the GPCR-regulated NF-κB pathway as a potential target for cancer therapy.

Going further upstream in the pathway, the first response in the signalling cascade for A3AR is modulation of cyclic AMP (cAMP) levels through regulation of adenylyl cyclase activity. We hence examined whether TMIGD3 i1 could cancel the effects of A3AR knockdown on cAMP levels in SJSA-1 cells (Fig. 6e). SJSA-1 cells downregulated for A3AR, along with overexpression of A3AR or TMIGD3 i1, were measured for cAMP levels following treatment with an A3AR-specific agonist CF102 (Cl-IB-MECA). We observed a dose-dependent decrease in cAMP levels on treatment with CF102 in control cells, which was not observed in cells downregulated for A3AR, although base levels of cAMP were increased regardless of CF102 treatment. Concomitant overexpression of A3AR cancelled the effects of A3AR knockdown and restored the dose-dependent response to CF102. Intriguingly, overexpression of TMIGD3 i1 in A3AR knockdown cells rescued the increased levels of cAMP to a certain level, but did not restore the dose-dependent response to CF102 (Fig. 6e). Western blotting revealed that levels of IκB increased following treatment with CF102 in control SJSA-1 cells. IκB levels were reduced on A3AR knockdown, which remained unchanged following treatment with CF102. Concomitant overexpression of A3AR restored reduced IκB levels by A3AR knockdown, with further increase in response to CF102. Consistent with the results in Fig. 6e, reduced IκB levels by A3AR knockdown were rescued by concomitant overexpression of TMIGD3 i1, but CF102 treatment failed to further increase IκB levels (Fig. 6f). In addition, we confirmed that knockdown of TMIGD3 or A3AR did not significantly alter levels of A3AR or TMIGD3, respectively, in SJSA-1 and Saos2 cells (Supplementary Fig. 6a,b). These results suggest that TMIGD3 i1 can substantially rescue the PKA−Akt−IκB−NF-κB axis but can only partially rescue cAMP levels induced by A3AR knockdown, suggesting a distinct function from A3AR. Taken together, our results indicate that TMIGD3 i1 suppresses malignant properties of OS via both overlapping and non-overlapping signalling pathways with A3AR.

## Discussion

TMIGD3 i1 is an uncharacterized protein whose function is completely unknown. The present study reveals a suppressive role for TMIGD3 i1 in malignant progression of OS. The functions of TMIGD3 i1 are largely mediated through inhibition of NF-κB activity via the PKA−Akt−IκB−NF-κB axis, similar to A3AR. In addition, our unbiased luciferase-based pathway analyses indicate distinct signalling pathways regulated by TMIGD3 from A3AR that may further contribute towards OS suppression. These include p53, Pax6, retinoic acid X receptor, AhR (aryl hydrocarbon receptor), GLI and progesterone receptor. Detailed studies questioning the significance of these pathways in OS progression and their relationship to TMIGD3 i1 or A3AR should be further elaborated on in the near future. Our initial screening did not identify A3AR as a candidate that suppressed sphere formation. This is probably because not all the shRNAs present in this library were validated at the time of purchase and the shRNAs for A3AR in the library might not be efficient enough to knock down A3AR.

Intriguingly, our data suggest that TMIGD3 i1 could also suppress cellular cAMP levels, as TMIGD3 i1 overexpression partially reduces cAMP levels induced by A3AR knockdown. However, considering that TMIGD3 i1 overexpression does not restore response to CF102 and TMIGD3 i1 does not have a typical GPCR structure, the observed reduction in cAMP levels by TMIGD3 i1 could be via its effect on other GPCRs including other adenosine receptors. It also remains unclear what can activate TMIGD3 i1 signalling, if adenosine could still be a ligand for TMIGD3 i1, and if TMIGD3 i1 could partially retain function as a GPCR. In addition, it would be important to characterize unappreciated roles of TMIGD3 i1 in the suppression of other types of cancer or immune-inflammatory diseases, similar to A3AR. Intriguingly, both TMIGD3 i1 and A3AR inhibit sphere-forming potential, which raises the possibility that they may also regulate stem-like properties of OS including tumour-initiating potential and self-renewability. Future studies are necessary to determine their roles in the regulation of stemness in OS.

Our data indicate that reduced expression of TMIGD3 is associated with OS progression. Moreover, we find that TMIGD3 i1, but not i3, plays roles in suppression of OS malignancy by overexpressing these isoforms. However, our generated PAb128 antibody detects both isoforms. Of note, it is challenging to generate antibodies that can exclusively identify TMIGD3 i1, as its N-terminal region is identical with A3AR, whereas its C-terminal region is identical with TMIGD3 i3. Generation of antibodies detecting a specific region in the splicing junction of exon A1 and exon T2 would be necessary to clearly discriminate TMIGD3 i1 from TMIGD3 i3 and A3AR. Nonetheless, our results suggest that protein expression of both TMIGD3 and A3AR in OS tissues is significantly lower than that in normal bone and lungs. Although the mechanisms behind reduced expression of these genes remain to be investigated, mRNA expression of TMIGD3 i1 and A3AR could be similarly regulated among different types of tissues or cancers, as transcription of these genes is likely to be driven by the same promoter. It would be important to determine the epigenetic and transcriptional regulation of these genes that could lead to its silencing specifically in cancer. In addition, future studies to determine expression patters of TMIGD3 i1 in different tissue types and cancers are required for revealing its role in various types of cancer. Interestingly, A3AR is shown to be overexpressed in some cancer types including prostate, colon and breast cancer^17^,^27^. Protein expression of TMIGD3 i1 might also be high in these cancers or could be regulated differently from A3AR. Moreover, expression of these proteins may be regulated in a cell type- and tissue context-dependent manner.

It should be noted that there is no significant difference in the expression of TMIGD3 and A3AR between primary and metastatic OS. This could be because expression of these genes may be lost during early stages of OS genesis. A caveat in this study is the lack of detailed patients' information to establish a correlation between expression of TMIGD3 or A3AR and clinical staging of the disease or prognosis, which should be determined as a future study.

According to cBioPortal (http://www.cbioportal.org/), there are cancer-associated mutations in the *TMIGD3* and *A3AR* genes. These mutations comprise missense and truncating mutations, the majority being missense mutations. These mutations are present throughout both the genes. Two hotspot mutations (V171I, R205Q) are detected in the *A3AR* gene, whereas no hotspot mutation is found in the *TMIGD3 i1* gene. Nonetheless, the functional significance of these cancer-associated mutations on OS progression remains to be investigated. It should be noted that *A3AR* knockout mice are not tumour prone and show an increased inflammatory response^28^. Thus, both A3AR and TMIGD3 i1 may not be typical tumour suppressors and rather be tumour modifiers. To examine the *in vivo* significance of A3AR, as well as TMIGD3 i1, on tumour progression, crossing knockout mouse for *A3AR* or *TMIGD3 i1* with a mouse model of cancer would be necessary.

Finally, agonists for A3AR including CF102 are currently under clinical trials for several diseases including hepatocellular carcinoma and rheumatoid arthritis^16^,^29^,^30^, whereas it would be necessary to discover compounds that activate TMIGD3 i1 signalling. However, of note, success of clinical trials may rely on the expression levels of TMIGD3 i1 and A3AR in tumours. As our study suggests low expression of TMIGD3 i1 and A3AR in OS tissues, the role of agonists is counter-intuitive. Hence, restoration of TMIGD3 i1 and A3AR expression levels in tumours is required while using their respective agonists. Alternatively, given that enhanced activity of NF-κB is implicated in increased chemoresistance and progression of multiple cancers including OS^31^,^32^,^33^, targeting NF-κB and its upstream effectors could be a promising therapeutic strategy for OS.

## Methods

### Cell lines

All the human OS cell lines including SJSA-1, U2OS, KHOS/NP, MG63 and Saos2 were purchased from ATCC and maintained in DMEM medium or Roswell Park Memorial Institute medium with 10% fetal bovine serum (FBS) and 1% penicillin–streptomycin in a humidified incubator at 37 °C with 5% CO_2_. The cell lines used in this study are not present in the database of commonly misidentified cell lines maintained by ICLAC. All cell lines were authenticated using STR profiling and tested negative for mycoplasma contamination.

### shRNAs and primers

A human whole-genome shRNA library was purchased from Open Biosystems (RHS4847, Decode RNAi-GIPZ: whole genome screening library-Positive selection kit). The following primers were used for PCR amplification of target shRNAs: forward primer (PRM 5023) and reverse primer (PRM 5035, Open Biosystems). To identify shRNAs, the following primer was used for sequencing: 5′-GCATTAAAGCAGCGTATC-3′.

The shRNAs/siRNAs used are as follows: *T6U*: 5′-AAGAACTAAGATCTTGAGATG-3′ (catalogue number VGH5518-200202459, GE Healthcare, Dharmacon Inc.), *T3*: 5′-TAGTTGCAGATGGCAGAAG (HSH003091-3-HIVmH1, Genecopoeia, Inc.), *A2a*: 5′-TTCTTCTGTGAGTGGTGAC (VGH5518-200180351), *A2b*: 5′-TGATGATAGATAAAGGCAG (VGH5518-200176914), *A2U:* 5′-TTGTCAGTAAGTCAACTAG (VGH5518-200223457, GE Healthcare, Dharmacon Inc.) and *ATa*: 5′-GCTTACCGTCAGATTCAGA (GE Healthcare, Dharmacon Inc.).

### Plasmids

Lentiviral vector encoding TMIGD3 i1 (accession number NM_020683) was purchased from GeneCopoeia (EX-Z4782-Lv152). This vector contains the entire coding sequence for *TMIGD3 i1* from start codon to stop codon without 3′-UTR, as well as hygromycin-resistant gene. The cDNA for A3AR and TMIGD3 i3 were purchased from Open Biosystems (MHS1010-7507630 Clone ID: 5176221, accession number BC029831; MHS1010-9206079 Clone ID: 5744223, accession number BC064411). The A3AR and TMIGD3 i3 cDNA without 3′-UTR were subcloned into pCDH-CMV-MCS-EF1-Puro (CD510B-1, SBI System Biosciences).

### Western blotting

Cells were directly lysed in 1.5 × SDS sample buffer and heated at 95 °C for 10 min, followed by loading onto 4–12% tris-glycine gel (Bio-Rad Laboratories), separated by electrophoresis and transferred to polyvinylidene fluoride membrane (GE Healthcare Life Sciences). Blots were incubated with primary antibodies for A3AR (sc-13938, clone H-80, dilution 1:200, Santa Cruz Biotechnology), TMIGD3 (PAb128, generated against aa235–aa248 CGIQRDFARDDMDF by GenScript, dilution 1:1,000), Flag (F3165, clone M2, dilution 1:2,000,Sigma-Aldrich), IκB (4814 S, clone L35A5, dilution 1:2,000, Cell Signaling), NF-κB p65 (8242S, clone D14E12, dilution 1:2,000, Cell Signaling), phosphorylated CREB (pCREB, S133; sc-101663, dilution 1:1,000, Santa Cruz Biotechnology), pAkt (S473; 4060S, clone D9E, dilution 1:1,000, Cell Signaling), Lamin B (sc-6216, clone C-20, dilution 1:1,000, Santa Cruz Biotechnology), glyceraldehyde 3-phosphate dehydrogenase (sc-25778, clone FL-335, dilution 1:2,000, Santa Cruz Biotechnology) and β-actin (A2228, clone AC-74, dilution 1:1,000, Sigma Aldrich) at 4 °C overnight. After washing with tris-buffered saline (TBS) plus 0.1% Tween 20, blots were incubated with appropriate secondary antibodies conjugated with fluorescence (IRDye; 650CW goat anti-rabbit IgG, dilution 1:5,000; 800CW goat anti-mouse IgG, dilution 1:10,000; 800CW donkey anti-goat IgG, dilution 1:5,000, Li-COR), followed by analysis with the Li-COR Odyssey infra-red imaging systems (Lincoln, Nebraska).

Protein extraction of cytoplasmic and nuclear proteins was performed using the Thermo Scientific NE-PER Nuclear and Cytoplasmic Extraction Reagents, according to the manufacturer's protocol. The nuclear and cytoplasmic extracts were suspended in SDS lysis buffer, followed by loading onto 4–12% tris-glycine gel. Uncropped original images of important blots are provided as a part of the Supplementary Information.

### Immunofluorescence

The cells were grown on poly-D-lysine/laminin-coated glass coverslips (BD Biosciences) in 24-well plates. Cells were fixed with 4% paraformaldehyde for 20 min and permeabilized with 0.3% Triton X-100 for 5 min. Following blocking in 1% BSA in PBS plus 0.3% Triton-X for 30 min at room temperature, cells were incubated with the following primary antibodies: p65 (8242S, clone D14E12, dilution 1:1,000, Cell Signaling), β-catenin (sc1496-R, clone C-18, dilution 1:1,000, Santa Cruz Biotechnology), p-Erk1/2 (4370S, clone D13.14.4E, dilution 1:250, Cell Signaling), IκB (4814S, clone L35A5, dilution 1:1,000, Cell Signaling) and p- p65 (S536; 3033S, clone 93H1, dilution 1:1,000, Cell Signaling) at 4 °C overnight. After washing with PBS, cells were incubated with fluorescence-conjugated secondary antibodies at room temperature for 1 h. Cells were mounted in ProLong Gold Antifade Reagent with DAPI (Invitrogen) and analysed using a Nikon epifluorescence microscope.

### Anchorage- and serum-independent growth assay

Cells (20 cells per well) were plated on 96-well ultra-low attachment plates (Corning Inc., Corning) in DMEM F12 medium containing 10 mM HEPES, 50 μM of putrescine, 20 nM of progesterone, ITS (insulin 25 mg ml^−1^, sodium selenite 25 μg ml^−1^ and transferrin 25 mg ml^−1^), epidermal growth factor (10 ng ml^−1^) and fibroblast growth factor (10 ng ml^−1^) for 10–14 days and numbers of spheres with sizes over 30 μm were counted. Sphere-forming potential was calculated as a percentage of number of spheres formed/number of cells seeded.

### Cell proliferation assay

Cells (1 × 10^4^) were seeded onto six-well plates (day 0). Live cell numbers were counted at days 2, 4, 6 and 8 following trypan-blue staining.

### Transwell migration assay

Migration assays were performed with 24-well transwell chambers (6.5 mm diameter, 8 μm pore size, Corning). Cells (5 × 10^3^ for SJSA-1, 1 × 10^4^ for KHOS/NP) in 100 μl of 0.5% FBS-containing DMEM were seeded on the upper chamber, whereas 10% FBS-containing DMEM was added in the lower chamber as a chemoattractant. Cells were allowed to migrate through the membrane for 10 h. The non-migrating cells were removed from the upper face of the filters and migrating cells to the lower face were stained with Diff-Quik Stain Set (Dade Behring). Stained cells in the entire fields were counted under an inverted microscope.

### Quantitative reverse transcription–PCR

RNAs isolated using the RNA-Quick MiniPrep (Zymo Research) was reversed transcribed to cDNA using M-MLV reverse transcriptase (Amresco), followed by TaqMan assays with ViiA7 (Life Technologies). The following assay numbers were used for probes: *cyclin D1* (HS00277039_m1), *cMyc* (HS00153408_m1), *IL-8* (HS00174103_m1) and *CXCL1* (HS00605382_gH, Life Technologies). The mRNA levels were normalized to those of *GAPDH* (Hs.PT.39a.22214836, Integrated DNA Technologies, Inc.).

### Measurement of cellular cAMP levels

Cells were seeded onto 12-well plates. Twenty four hours later, cells were incubated with serum-free (DMEM/F12 with 10 mM HEPES) media for 15 min. Cells were then incubated with adenosine deaminase (3 units per ml) followed by treatment with CF102 (Cl-IB-MECA, Sigma Aldrich) at different concentrations and incubated for an additional 10 min. The cells were harvested and lysed, to measure cAMP levels according to the manufacturer's protocol (STA-500, Cell Biolabs Inc.).

### Signal analysis and luciferase assay

Cignal 45-Pathway Reporter Array was purchased from SABiosciences (CCA-901L) and luciferase assays were performed according to the manufacturer's protocol. Briefly, 50 μl Opti-MEM was added to each well of the Cignal Finder Array plate coated with reporter assay constructs. Subsequently, 50 μl of Opti-MEM containing 0.3 μl of Attractene Transfection Reagent (Qiagen) was used for each individual transfection. Following a 20 min incubation, 50 μl of a cell suspension containing 1–3 × 10^4^ cells in Opti-MEM with 10% of fetal bovine serum and 1% non-essential amino acids (NEAA) was added to each well. After 16 h of transfection, the medium was changed to complete growth medium and further incubated for 36 h, followed by luciferase assays using the Dual-Luciferase Reporter Assay System (Promega). To measure activities of NF-κB and β-catenin, we performed luciferase assays following transfection of cells with luciferase reporter plasmids encoding promoters responsive to NF-κB (pGL4.32[luc2P/NF-κB-RE/Hygro]) or TCF/LEF (TOPFlash), along with the pRL-TK (Promega) plasmid as an internal control, using the JetPRIME transfection reagent (Polyplus transfection). The dual reporter luciferase assay system (Promega) was used to measure promoter activities 36 after transfection, according to manufacturer's instructions. The values of luciferase activities were determined using a luminometer (BioTek Synergy H4) and were normalized to the internal control. All transfection experiments were repeated at least three independent times.

### Mice and *in vivo* tumor formation assay

All mice were maintained under specific pathogen-free conditions and experimental procedures were performed according to the institutional animal welfare guidelines approved by Institutional Animal Care and Use Committee for the University of Kansas Medical Center.

For subcutaneous tumour growth assays, cells were dissociated into single-cell suspensions using non-enzymatic cell dissociation solution (Sigma Biochemicals) and numbers of live cells were counted following trypan blue staining (Thermo Fisher Scientific). Cell suspension in 50 μl of 4.5 mg ml^−1^ Matrigel (BD Biosciences) in Hank's balanced salt solution was subcutaneously injected into flanks of NIH-III nude mice (Charles River). Tumours were measured three dimensionally two to three times a week for 17–20 days. For tail vein assays, 150 μl of cell suspension (5 × 10^4^) was injected into the lateral veins of nude mice. Mice were monitored for laboured breathing and the numbers of pulmonary tumour nodules were evaluated 6 weeks after injections. For orthotopic injections, 15 μl of cell suspension (1 × 10^5^) was injected into femoral bone marrow space of anaesthetized NOD-scid IL2Rγ^null^ mice (The Jackson Laboratories)^22^. When the tumours reached ∼1 cm in thigh diameter, the mice were killed. The weight of the primary tumours and numbers of tumour nodules in the lungs and liver (>0.5 mm) were measured.

### IHC for human tissues

Formalin-fixed paraffin-embedded tissues of 16 primary and 17 metastatic OS, as well as 10 normal lung tissues, were provided by the KUMC histology core facility. All the samples were anonymous and no patient information was given. All samples were collected during surgery for biopsy from patients with informed consent, admitted at the University of Kansas Medical Center (KUMC), and the research was approved by the institutional review board (IRB) of KUMC^34^. We also purchased a tissue microarray (OS804a, US Biomax) consisting of 38 primary OS and 10 normal bone tissues.

Sections (4 μm thick) from the aforementioned tissues were deparaffinized in xylene, rehydrated in grades of alcohol, rinsed in tap water and blocked with 0.3% hydrogen peroxide for 30 min. Antigen retrieval was performed in a steamer with sodium citrate buffer (10 mM sodium citrate, pH 6.0) for 20 min. After blocking in 2.5% normal horse serum for 30 min, sections were incubated with rabbit anti-human TMIGD3 (PAb128, dilution 1:500, generated by GenScript) and A3AR (A3R32-A, dilution 1:500, Alpha Diagnostics) antibodies for 30 min at room temperature. After washing in PBS, sections were incubated in anti-rabbit biotinylated secondary antibody for 30 min. The signal was detected using the Vectastain Elite ABC kit (Vector Laboratories). Pre-immune serum and normal rabbit IgG (Vector Labs, Burlingame) were used as negative controls. Two independent investigators evaluated the stained sections. Scoring was based on intensity and extensity. The scoring was determined by assessing the whole tumour section and each sample was scored on a scale of 0–3 for extensity with 0 corresponding to <25% of positive tumour cells, 1 for 26–50%, 2 for 51–75% and 3 for 76–100%. The intensity of immunostaining was determined as 0 (negative staining), 1 (weakly positive staining), 2 (moderately positive staining) and 3 (strongly positive staining). The immunoreactive score of each section was calculated by the sum of these two parameters and presented as a score ranging between 0 and 6.

### Statistical analyses

Proliferation assays, subcutaneous tumour growth assays and expression of protein in tissues were analysed using analysis of variance as indicated in the figure legends. All other analyses were done using Student's *t*-test (two-tailed). All analyses were performed using GraphPad Prism software (GraphPad Software, Inc.). The sample size was not chosen based on any statistical methods. All the experiments were performed at least three independent times. The number of animals used for the experiment is provided in the corresponding figure legend. Appropriate statistical analyses were chosen based on the data distribution. The experiments were not randomized and no samples were excluded. The investigators were not blinded before the experiment or during outcome assessment. Panels in Figs 5a–c,f–h and 6c,d,f show a representative image of at least three independent experiments.

### Data availability

The authors declare that the data supporting the findings of this study are available within the paper and its Supplementary Information files or available from the corresponding author upon reasonable request.

## Additional information

**How to cite this article:** Iyer, S. V. *et al*. Genome-wide RNAi screening identifies TMIGD3 isoform1 as a suppressor of NF-κB and osteosarcoma progression. *Nat. Commun.* **7,** 13561 doi: 10.1038/ncomms13561 (2016).

**Publisher's note**: Springer Nature remains neutral with regard to jurisdictional claims in published maps and institutional affiliations.

## Supplementary Material

Supplementary Information Supplementary Figures 1-7 and Supplementary Tables 1-2.
