# Supplementary material for: Genome-wide RNAi screening identifies TMIGD3 isoform1 as a suppressor of NF-κB and osteosarcoma progression
Source: Nat Commun. 2016 Nov 25;7:13561. doi: 10.1038/ncomms13561 (PMC5133659; doi:10.1038/ncomms13561)
Supplement: Supplementary Information — Supplementary Figures 1-7 and Supplementary Tables 1-2. [file ncomms13561-s1.pdf]

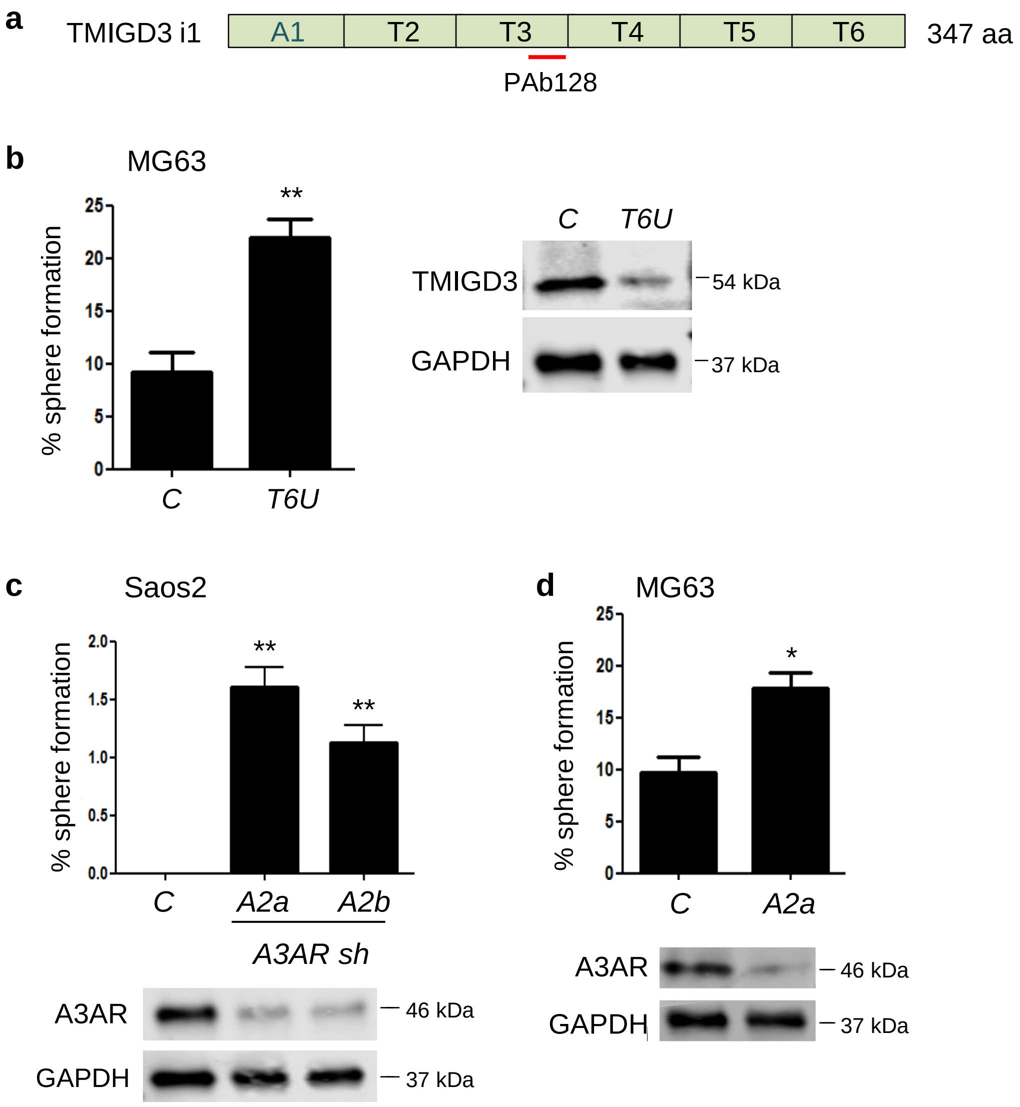

Supplementary Figure 1 | Knockdown of TMIGD3 or A3AR increases sphere formation in multiple OS cells.
(**a**) TMIGD3 i1 protein and location of the TMIGD3-specific antibody (PAb128, red bar). (**b**) Sphere formation assays using MG63 cells infected with lentiviral vectors encoding non-silencing *control* (*C*) or *TMIGD3* (*T6U*) shRNAs (left). Error bars: means ± S.D. (n=3 independent experiments). ** $p<0.01$; Student's t-test (two-tailed). Representative images of western blotting for TMIGD3 and GAPDH (right). (**c, d**) Sphere formation assays using Saos2 (**c**) and MG63 (**d**) cells downregulated for A3AR (*A2a*, *A2b*). Graphs show percentage of sphere formation. Representative images of western blotting for A3AR and GAPDH are present below the graph. Error bars: means ± S.D. (n=3 independent experiments). * $p<0.05$, ** $p<0.01$; Student's t-test (two-tailed).

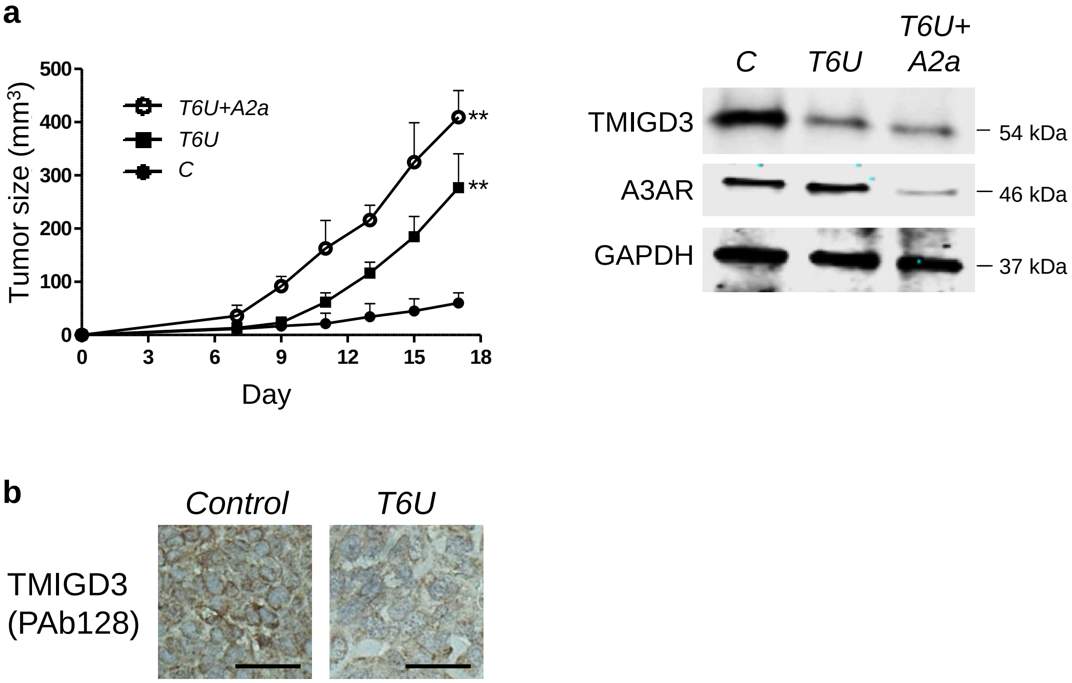

Supplementary Figure 2 | Downregulation of TMIGD3 and A3AR is associated with OS malignancy *in vivo* and clinically.
(**a**) Tumor growth of SJSA-1 cells downregulated for TMIGD3 alone (*T6U*), or in combination with A3AR (*T6U+A2a*). Cells ($1x10^6$) were subcutaneously injected into nude mice, and sizes of tumors were measured twice a week for 17 days. Error bars: means ± S.D. (n=5 mice per group). ** p<0.01; Two-way ANOVA. Representative images of western blotting for TMIGD3, A3AR, and GAPDH (right). (**b**) IHC for TMIGD3 with PAb128 using SJSA-1-derived tumors infected with lentiviral vectors encoding non-silencing (*control*) or *TMIGD3* (*T6U*) shRNAs. Scale bar: 100 µm.

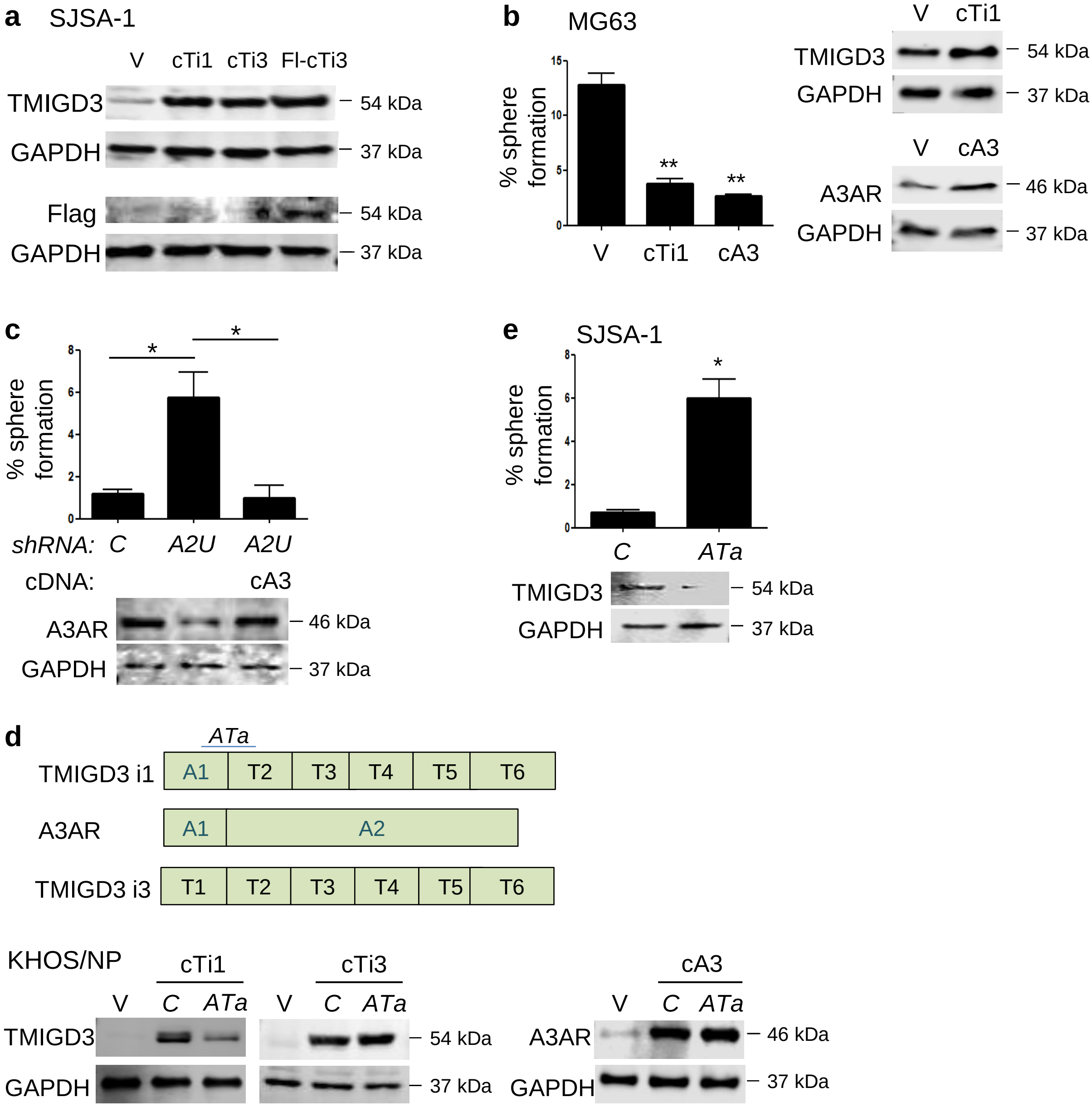

Supplementary Figure 3 | Manipulation of TMIGD3 and A3AR expression and its effects on sphere formation.

(**a**) TMIGD3 i1 and i3 run at a similar size. Representative images of western blotting using SJSA-1 cells expressing vector control (V), TMIGD3 i1 (cTi1), TMIGD3 i3 (cTi3), or Flag-tagged cTi3 (Fl-cTi3) for indicated proteins. (**b, c**) Sphere formation assays using MG63 cells overexpressing vector control (V), TMIGD3 i1 (cTi1), or A3AR (cA3, **b**) and SJSA-1 cells following knockdown of A3AR with a shRNA targeting the 3' UTR (*A2U*) and concomitant overexpression of A3AR (*A2U* + cA3) (**c**). Graph showing % of sphere formation. Representative images of western blotting for TMIGD3, A3AR, and GAPDH. Error bars: means ± S.D. (n=3 independent experiments). * $p<0.05$, ** $p<0.01$; Student's t-test (two-tailed). (**d**) Design of the *ATa* siRNA that targets only TMIGD3 i1 with minimal effects on TMIGD3 i3 and A3AR. Immunoblots for TMIGD3 and A3AR using KHOS/NP cells overexpressing TMIGD3 i1 (cTi1), TMIGD3 i3 (cTi3), or A3AR (cA3) following transfection of *control* or *ATa* siRNAs. (**e**) Sphere formation assays using SJSA-1 cells following transfection of the *ATa* siRNA. Representative immunoblots showing knockdown of TMIGD3 (below). Error bars: means ± S.D. (n=3 independent experiments). * $p<0.05$; Student's t-test (two-tailed).

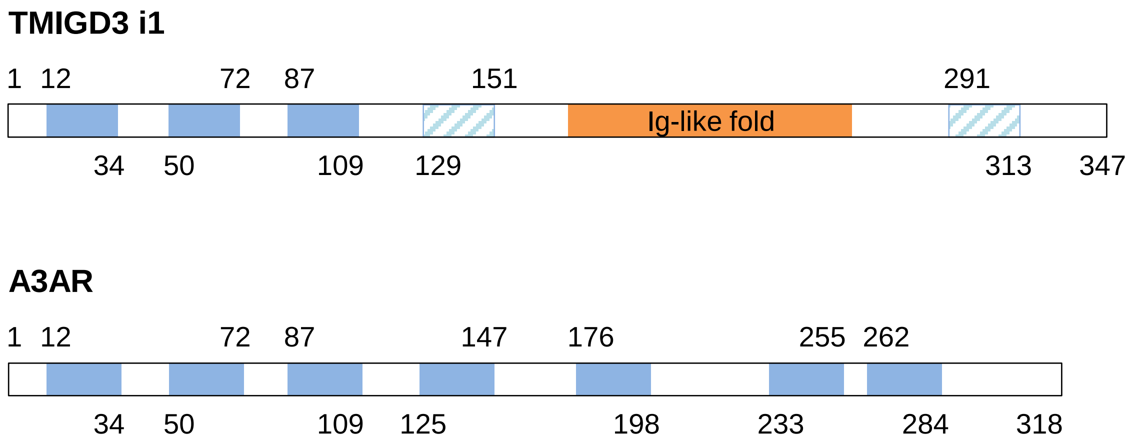

Supplementary Figure 4 | Predicted structure of TMIGD3 i1 and its comparison with A3AR.

Blue filled boxes indicate reported transmembrane helices. Blue boxes with oblique lines indicate predicted transmembrane helices. Three N-terminal transmembrane helices are common between TMIGD3 i1 and A3AR. Orange box indicates the Ig-like fold. Numbers indicate amino acid locations.

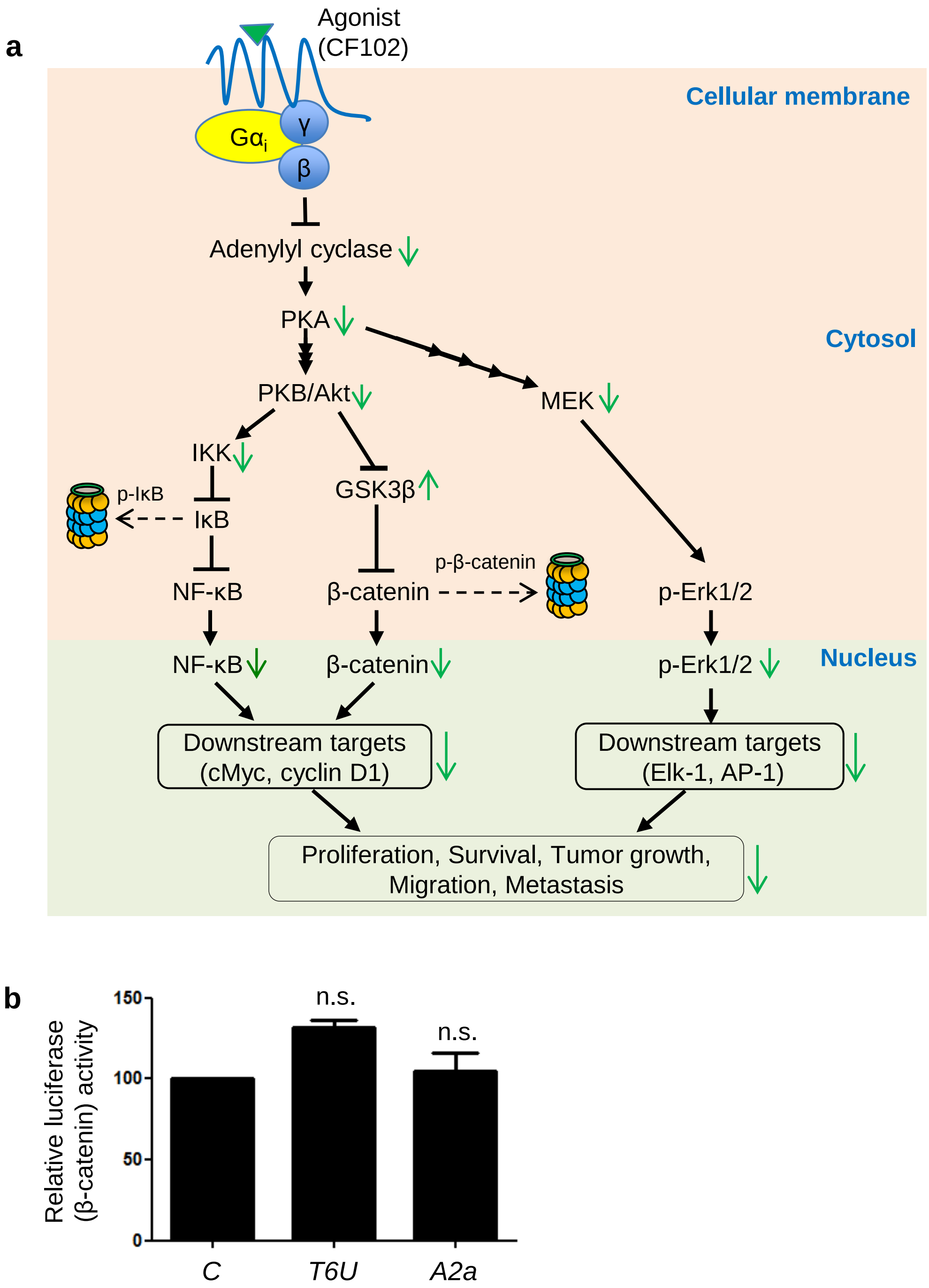

Supplementary Figure 5 | Signaling pathways regulated by A3AR and effects of knockdown of TMIGD3 or A3AR on the β-catenin activity. (**a**) Cancer associated-signaling pathways regulated by A3AR. (**b**) TOPFlash luciferase reporter assays for measuring the β-catenin activity in SJSA-1 cells downregulated for TMIGD3 (*T6U*) or A3AR (*A2a*). Graph showing relative luciferase activity normalized to that of SJSA-1 cells infected with non-silencing *control* (*C*) lentiviral vector. Error bars: means ± S.D. (n=3 independent experiments). n.s.: not significant; Student's t-test (two-tailed).

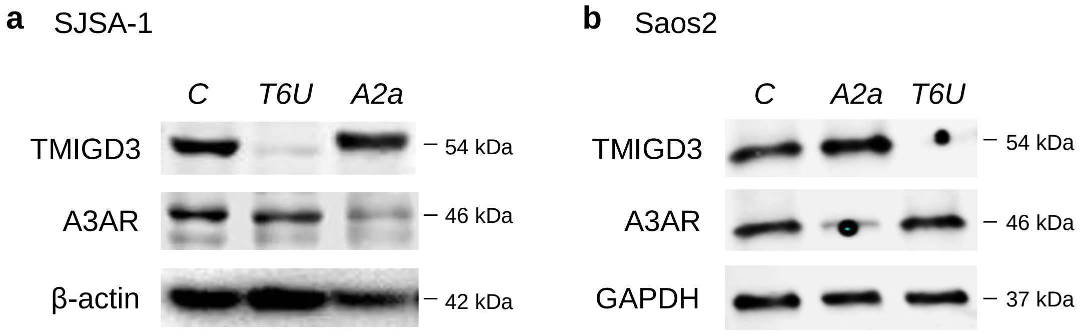

Supplementary Figure 6 | Knockdown of TMIGD3 (*T6U*) or A3AR (*A2a*) does not alter protein levels of A3AR or TMIGD3, respectively.
(**a, b**) Representative images of western blotting for TMIGD3, A3AR, β-actin, and GAPDH in SJSA-1 (**a**) and Saos2 (**b**) cells downregulated for TMIGD3 (*T6U*) or A3AR (*A2a*).

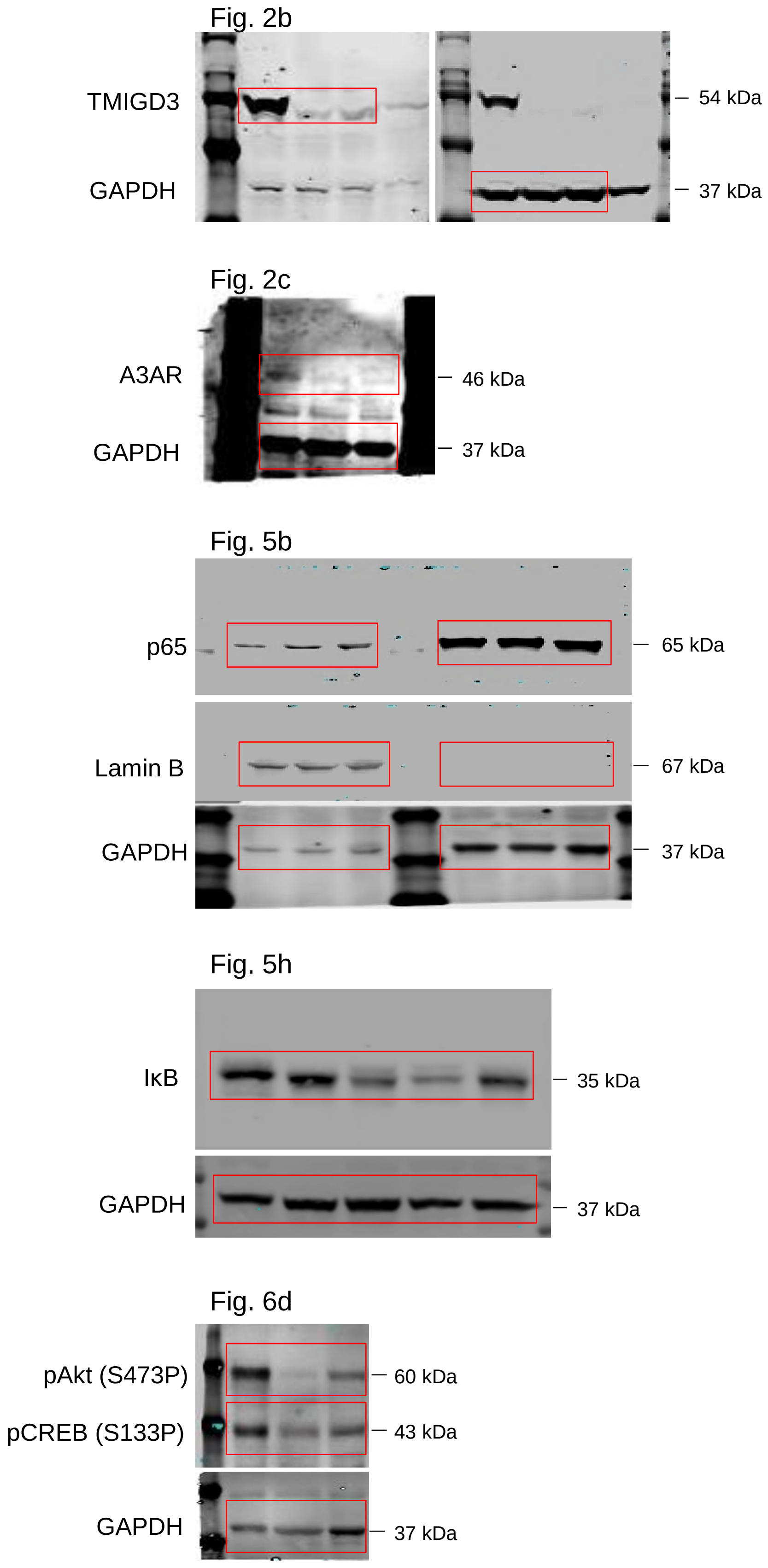

Supplementary Figure 7 | Uncropped images of important western blotting presented in the main figures.
Red boxes indicate the areas presented in the representative figures.

Supplementary Table 1 | Sphere forming potential of OS cell lines

Cell line	Total cell # examined	% sphere formation* (average +/- S.D.) 30-75 μm, >75 μm
U2OS	140,000	0+/-0, 0+/-0
Saos2	140,000	0+/-0, 0+/-0
SJSA-1	140,000	0.5+/-0.5, 0+/-0
MG63	2,160	7.7+/-1.5, 4.5+/-2.1
KHOS/NP	1,780	9.2+/-2.1, 7.7+/-1.8

Spheres < 30 μm in diameter were not counted as spheres.
* % sphere formation: a percentage of # of spheres formed/# of cells seeded.

Supplementary Table 2 | Functions of candidates

Gene name (abbreviation)	Function
Transmembrane and immunoglobulin domain containing 3 (TMIGD3)	Unknown. Ig-like fold containing protein family. TMIGD3 i1 is an alternative splicing form of adenosine A3 receptor (A3AR), whereas TMIGD3 i3 has no overlap with A3AR.
Solute carrier family 45-4 (SLC45A4)	Unknown. No reference.
Spi-B (Spi-B)	Ets family transcription factor implicated in B cell lymphoma suppression.
Glutamate receptor interacting protein 1 (GRIP1)	Cytoplasmic multi-PDZ scaffolding protein implicated in neuronal synaptic function.
Zinc finger RNA binding protein 2 (ZFR2)	Unknown. No reference.
Spermatogenesis and centriole associated 1 (SPATC1)	Centrosomal protein implicated in zygotic cell division (Y chromosome).
Periphilin-1 (PPHLN1)	Gastric cancer antigen Ga50 involved in epithelial differentiation.
